# Diffusion-weighted magnetic resonance imaging using a preclinical 1 T PET/MRI in healthy and tumor-bearing rats

**DOI:** 10.1186/s13550-019-0489-6

**Published:** 2019-02-22

**Authors:** Jakob Albrecht, Dietrich Polenz, Anja A. Kühl, Julian M. M. Rogasch, Annekatrin Leder, Igor M. Sauer, Magor Babos, Gabor Mócsai, Nicola Beindorff, Ingo G. Steffen, Winfried Brenner, Eva J. Koziolek

**Affiliations:** 1 Department of Nuclear Medicine, Charité – Universitätsmedizin Berlin, Augustenburger Platz 1, 13353 Berlin, Germany; 2 German Cancer Consortium (DKTK), Im Neuenheimer Feld 280, 69120 Heidelberg, Germany; 3 German Cancer Research Center (DKFZ) Heidelberg, Im Neuenheimer Feld 280, 69120 Heidelberg, Germany; 4 Berlin Experimental Radionuclide Imaging Center (BERIC), Charité - Universitätsmedizin Berlin, Südstraße 3, 13353 Berlin, Germany; 5 iPATH.Berlin – Immunopathology for Experimental Models, Charité – Universitätsmedizin Berlin, Berlin Institute of Health, Core Unit, Hindenburgdamm 30, 12203 Berlin, Germany; 6 Department of Surgery, Campus Charité Mitte, Luisenstraße 64, 10117 Berlin, Germany; 7 Department of Surgery, Campus Virchow Klinikum, Charité - Universitätsmedizin Berlin, Mittelallee 4, 13353 Berlin, Germany; 8 Mediso Medical Imaging Systems, Laborc utca 3, Budapest, 1037 Hungary

**Keywords:** DWI, ADC, Tumor microenvironment, Hepatocellular carcinoma, Preclinical, 1 Tesla, PET/MRI, Rats

## Abstract

**Background:**

Hybrid positron emission tomography and magnetic resonance imaging (PET/MRI) scanners are increasingly used for both clinical and preclinical imaging. Especially functional MRI sequences such as diffusion-weighted imaging (DWI) are of great interest as they provide information on a molecular level, thus, can be used as surrogate biomarkers. Due to technical restrictions, MR sequences need to be adapted for each system to perform reliable imaging. There is, to our knowledge, no suitable DWI protocol for 1 Tesla PET/MRI scanners. We aimed to establish such DWI protocol with focus on the choice of *b* values, suitable for longitudinal monitoring of tumor characteristics in a rat liver tumor model.

**Material and methods:**

DWI was first performed in 18 healthy rat livers using the scanner-dependent maximum of 4 *b* values (0, 100, 200, 300 s/mm^2^). Apparent diffusion coefficients (ADC) were calculated from different *b* value combinations and compared to the reference measurement with four *b* values. T2-weighted MRI and optimized DWI with best agreement between accuracy, scanning time, and system performance stability were used to monitor orthotopic hepatocellular carcinomas (HCC) in five rats of which three underwent additional 2-deoxy-2-(^18^F)fluoro-d-glucose(FDG)-PET imaging. ADCs were calculated for the tumor and the surrounding liver parenchyma and verified by histopathological analysis.

**Results:**

Compared to the reference measurements, the combination *b* = 0, 200, 300 s/mm^2^ showed the highest correlation coefficient (*r*_s_ = 0.92) and agreement while reducing the acquisition time. However, measurements with less than four *b* values yielded significantly higher ADCs (*p* < 0.001). When monitoring the HCC, an expected drop of the ADC was observed over time. These findings were paralleled by FDG-PET showing both an increase in tumor size and uptake heterogeneity. Interestingly, surrounding liver parenchyma also showed a change in ADC values revealing varying levels of inflammation by immunohistochemistry.

**Conclusion:**

We established a respiratory-gated DWI protocol for a preclinical 1 T PET/MRI scanner allowing to monitor growth-related changes in ADC values of orthotopic HCC liver tumors. By monitoring the changes in tumor ADCs over time, different cellular stages were described. However, each study needs to adapt the protocol further according to their question to generate best possible results.

**Electronic supplementary material:**

The online version of this article (10.1186/s13550-019-0489-6) contains supplementary material, which is available to authorized users.

## Introduction

Providing information on a molecular level, diffusion-weighted magnetic resonance imaging (DWI) has been proven to be a useful parameter for the differentiation between healthy and pathologically altered tissue. In fact, DWI has been used for tumor tissue characterization and early assessment of tumor therapy response since almost two decades in preclinical and clinical studies [1–7] (reviewed in [8]). DWI is also sensitive to inflammatory processes, e.g., in the lung [9], intestine [10], and liver [11].

DWI is a quantitative imaging technique that allows the measurement of thermally driven molecular movement of water, also known as Brownian motion. While water molecules move freely in pure water, the magnitude of the range of motion in body tissues depends on its composition, i.e., cell density and extracellular matrix, and so reflects the characteristics of tissue types (reviewed in [12]). At a steady state, the apparent diffusion coefficient (ADC) as a measure for DWI is approximately 3.0 mm^2^/s for pure water preheated to 37 °C [13]. Stejskal and Tanner first introduced a way for diffusion-sensitive magnetic resonance imaging (MRI) in 1965 [14] by adding two diffusion gradients to a common MRI sequence, such as spin-echo (SE) or echo-planar-imaging (EPI), to measure the signal attenuation caused by the motion of a water molecule. For quantitative analysis, DWI enables the calculation of the ADC, which is mainly dependent on the combination of diffusion weighting factors, the so-called *b* values. However, the optimal magnitude and amount of *b* values remain controversial as the available hardware [15], the organ of interest [16–18], and the method of data analysis [19] play a major role for this technique.

In preclinical research, much effort has been put into the development of hybrid imaging devices such as the nanoScan positron emission tomography/magnetic resonance imaging (PET/MRI) scanner (Mediso, Hungary). The MRI part of this dedicated 1 Tesla (T) small animal imaging system is based on a permanent magnet allowing to operate the scanner in a standard animal research laboratory without the need of extra cooling or shielding of the MR compound [20]. However, due to technical restrictions of the 1 T field strength, imaging protocols and MRI sequences established for higher field strength scanners cannot be simply transferred to this scanner, e.g., DWI is only available with SE sequences that are limited to a maximum of four *b* values with a maximum magnitude of 600 s/mm^2^ depending on the chosen parameters. For achieving both reasonable imaging quality and reliable quantitative data sets, functional MR imaging such as DWI needs to be adapted and optimized for this device to achieve sufficient and valid data while keeping scanner performance and scan duration and, thus, duration of anesthesia, at a reasonable level.

The aim of this study was to evaluate the feasibility of DWI scanning with the Mediso stand-alone 1 T nanoScan PET/MRI equipped with a permanent magnet and no additional cooling for longitudinal animal studies. Because there is, to the best of our knowledge, no published study proposing a protocol for DWI for this system, we first established an imaging protocol for DWI in the abdominal region for the liver in healthy rats, focusing on the selection of *b* values for ADC calculation. In a second step, we applied the optimized DWI protocol in a pilot study to monitor biologic alterations of the liver in a hepatocellular carcinoma (HCC) homograft tumor model by hybrid 2-deoxy-2-(^18^F) fluoro-d-glucose (FDG) PET/MR imaging.

## Materials and methods

### Cell culture

Native rat HCC cell line N1-S1 was purchased from the American Type Culture Collection (ATCC). N1-S1 cells were maintained in RPMI1640 medium supplemented with 10% fetal calf serum and 1% penicillin/streptomycin. Cell cultures were grown in roller bottles at 37 °C in an atmosphere of room air supplemented with 5% CO_2_. Medium was changed to antibiotic-free medium 48 h prior to in vivo cell inoculation.

### Generation of the HCC tumor homograft

All animal experiments were performed in accordance with national and local guidelines for animal welfare and were approved by the animal ethics committee of the state Berlin (LAGeSo, Reg. No. G0059/14).

For in vivo experiments, Sprague-Dawley rats where orthotopically inoculated into the liver with N1-S1 cells. After achieving sufficient depth of anesthesia by Isoflurane and low dose of Ketamine (10 mg/kg), a small median laparotomy was performed, and the left lateral liver lobe was exposed. Cells were injected at a very slow rate by visualization directly under the hepatic capsule. Successful inoculation was verified by a pale, whitish area around the point of injection underneath the hepatic capsule. Animals were maintained under pathogen-free conditions in a 12-h day and night cycle and were fed with sterile food and water ad libitum. Rats were weight-monitored during the in vivo experiments at least once a week.

### Phantom study

To pre-validate DWI by a phantom study, three water phantoms were included in the analysis: one was filled with 2 ml pure water, the others with 5% and 10% agarose in 2 ml water, respectively. All phantoms were preheated to 37 °C and temperature maintained by placing them on the heated animal bed. DWI was carried out using *b* = 0, 200, 400, 600 s/mm^2^ and *b* = 0, 100, 200, 300 s/mm^2^ with no respiratory gating resulting in an acquisition time of approximately 5 min.

### In vivo imaging

Imaging was performed on a small animal 1 T PET/MRI (nanoScan PET/MRI, Mediso, Hungary) equipped with a 1 T permanent magnet and a gradient coil providing amplitudes up to 450 mT/m. A dedicated rat whole-body coil was used for RF-transmission and signal receiving. Rats were anesthetized with general anesthesia (1–2% isoflurane—3% for initiation—with oxygen at a flow rate of approximately 0.5 l/min). Body temperature was maintained at 37 °C during the time of imaging by using a heated bed aperture. A pressure-sensitive pad was placed under the thorax and used for respiratory monitoring and gating.

DWI of the healthy liver was performed in healthy Sprague-Dawley rats (*n* = 18). An axial T1 2D SE sequence was acquired for correct anatomical delineation of the liver. DWI was carried out using an SE sequence with respiratory gating with the following parameters: TR = 362–560 ms; TE = 14.6 ms; slice thickness/gap = 1.3 mm/0.2 mm; matrix = 160 × 160 mm; diffusion weighting direction = *x*, *y*, *z*; and number of excitations = 1. *b* values were chosen due to the results of the phantom studies: 4b = 0, 100, 200, 300 s/mm^2^. This resulted in an acquisition time of 42 min at a respiratory rate of 40/min.

For tumor growth monitoring and DWI in HCC-bearing rats (*n* = 5), animals were scanned before and at three time points between days 10 and 20 after tumor cell inoculation. For tumor delineation and volumetry, a T2w 2D fast spin echo (FSE) sequence was established: TR = 7500 ms; TE = 90 ms; slice thickness/gap = 1.3 mm/0.2 mm; matrix = 258 × 228 mm; external averages = 5; and number of excitations = 2. To visualize surrounding liver parenchyma, a T1 3D gradient echo (GRE) sequence was used: TR = 50 ms; TE = 2.3 ms; flip angle = 40°; slice thickness/gap = 0.5 mm/0 mm; matrix = 160 × 160 mm; and number of excitations = 2. DWI in HCC homograft rats was performed under respiratory gating, and the chosen *b* values (*b* = 0, 200, 300 s/mm^2^) resulted in a mean acquisition time of 29 min at a respiratory rate of 40/min.

As a proof of concept, additional PET imaging was performed in *n* = 3 HCC homograft rats to monitor glucose metabolism during tumor growth at three time points after inoculation of the tumor cells, i.e., after 10 and 20 days as well as either 13 or 16 days. Due to tracer availability, one animal was imaged at two time points only. FDG was injected intravenously (iv) into the rat tail vein (0.2 ml; approximately 30 MBq) and scanned after 30 min to ensure tracer accumulation in the tumor tissue (iterative image reconstruction, [ordered subset expectation maximization]; iterations, 6; subsets, 6; voxel size, 500 × 500 × 600 μm^3^). Acquisition protocols included a material map for attenuation and scatter correction during PET reconstruction. Prior to imaging, the PET/MRI system was calibrated for FDG so that local tissue concentrations in the tumor could be measured quantitatively in kBq/ml tissue.

### Image analysis: MRI

Image analysis was conducted with InterView Fusion 3.01 (Mediso, Hungary).

ADC maps were automatically calculated on a pixel by pixel basis. The image pixel intensity depends on the *b* value according to the following equation:

$$ I={I}_0{e}^{- bD} $$

where *D* is the diffusion coefficient of tissues. The natural logarithms of the measured intensities are plotted against the *b* values. The slope of the regression line represents the ADC.

For protocol establishment and optimization, ADC values were calculated from the following *b* value combinations: 4b (*b* = 0, 100, 200, 300 s/mm^2^), 3b1 (*b* = 0, 100, 200 s/mm^2^), 3b2 (*b* = 0, 200, 300 s/mm^2^), and 2b (*b* = 0, 300 s/mm^2^). These combinations were compared with each other with regard to accuracy, stability of the scanner performance, and acquisition time.

To correctly delineate liver parenchyma and tumor, ADC maps were fused with T1 and T2 images, respectively. For selection of healthy liver parenchyma, a region of interest (ROI) was manually drawn onto four subsequent slices with the most liver parenchyma visible, i.e., slices directly adjacent to the recessus costodiaphragmaticus. In tumor-bearing animals, ROIs for ADC mapping of the surrounding healthy liver parenchyma were placed onto four subsequent slices around the largest diameter of the tumor. The tumor ADC was calculated slice by slice for the whole tumor. Slices showing strong motion artifacts despite respiratory gating were excluded from ADC calculation.

ADC maps from water and agarose phantoms were generated from four subsequent slices.

Total tumor volumes were calculated based on T2-weighted images by adding up all tumor-related voxels.

### Image analysis: PET

Analysis was performed with dedicated software (ROVER, version 3.0.34, ABX advanced biochemical compounds GmbH, Radeberg, Germany). FDG-avid tumor tissue (Vol_vital_) was delineated with a unified fixed relative threshold at 45% of the maximum activity in kBq/ml in the tumor without background adaptation and defined as “viable.” If necessary, manual corrections based on MRI were performed to exclude adjacent non-tumorous FDG uptake in surrounding tissue. To calculate the percentage of viable tumor tissue, the volume of the FDG-avid tumor tissue (PET: Vol_vital_) as well as the whole tumor volume (T2w: Vol_tumor_) were determined. For correct delineation of the whole tumor, fused FDG-PET/T2w images were used.

### Histopathology of tumor surrounding liver parenchyma

Liver tissue surrounding the tumors (left liver lobe), as well as healthy liver were snap frozen for immunohistochemistry. Cryo sections (approx. 5 μm) from snap frozen tissues were cut and air-dried overnight. For histochemistry, sections were fixed with ice-cold aceton p.a. and stained with hematoxylin and eosin (H&E). For immunohistochemistry, sections were fixed in 4% formalin, dehydrated in ethanol, and air-dried prior to heat-induced epitope retrieval. Sections were rinsed with running tap water and incubated with antibodies directed against CD3ε (Dako #IR50361–2), MPO (clone MPO7, Dako), or CD163 (clone EPR19518, Abcam) at room temperature. This was followed by incubation with secondary antibodies (biotinylated donkey anti-rabbit; Dianova). Biotin was detected by streptavidin conjugated with alkaline phosphatase, which converted the substrate solution into dye deposits of FastRed (both Dako). Primary antibodies were omitted in negative control sections.

Stained sections were analyzed in a blinded fashion using an AxioImager Z1 (ZEISS) and a Vectra3 (PerkinElmer) imager, respectively. For quantification of positive cells or total hepatocytes in 10 high power fields (field of vision with highest magnification; × 400 and × 200, respectively), inForm software (PerkinElmer) was used.

### Statistical analysis

Statistical analysis was conducted with IBM SPSS Statistics version 24. Due to small sample size, normal distribution was not assumed and in consequence data were described using median, interquartile range (IQR), minimum and maximum values, and depicted as boxplots. Paired groups were compared using non-parametric Wilcoxon test. All tests were two-sided and the level of significance was set to *p* < 0.05. Calculations of the ADC values using less *b* values (3b2, 3b1, 2b) were compared to values calculated with the maximum of four *b* values (4b = 0, 100, 200, 300 s/mm^2^) considered as the reference standard of this scanner. Correlations are shown using spearman’s rank correlation coefficient. Agreement of the different ADC calculation methods and scattering was evaluated as described by Bland et al. [21].

## Results

### Phantom study

Phantom studies with 37 °C pre-heated water phantoms revealed a mean ADC of 2.8 mm^2^/s for *b* = 0, 100, 200, 300 s/mm^2^ and 2.6 mm^2^/s for *b* = 0, 200, 400, 600 s/mm^2^. As expected, agarose phantoms showed decreasing ADC values with increasing agarose concentration (Table 1).

**Table 1:** ADC values of 37 °C pre-heated water and water/agarose phantoms

*b* value (s/mm^2^)	Phantom	ADC (mm^2^/s)
0, 100, 200, 300	Water	2.840
	5% agarose	2.627
	10% agarose	2.156
0, 200, 400, 600	Water	2.594
	5% agarose	2.328
	10% agarose	1.511

### DWI-protocol optimization

To establish a DWI protocol in areas of high respiratory motion (liver and abdominal region) while keeping the scanning time at a moderate level with respect to animal anesthesia and animal throughput as well as long-term scanner performance stability during a workday, *b* = 300 was chosen as the highest *b* value. DWI was first performed in healthy rat livers using the maximum of four *b* values (*b* = 0, 100, 200, 300 s/mm^2^) for this scanner as the reference setting.

**Fig. 1:**
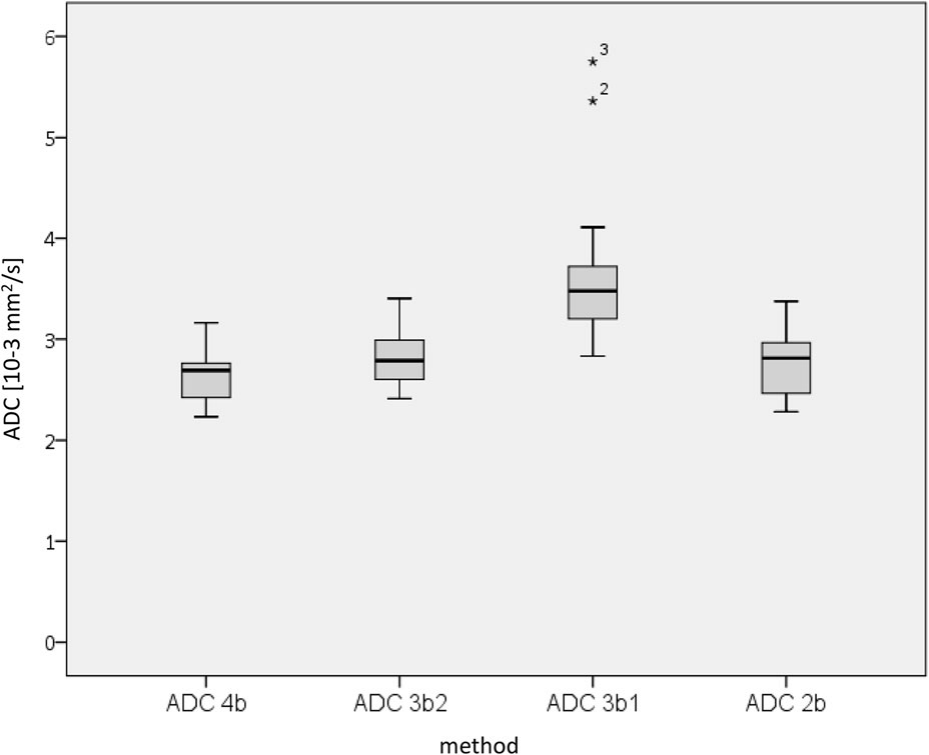
Boxplots of absolute ADC values calculated from different *b* value combinations: 4b = 0, 100, 200, 300 s/mm^2^, 3b2 = 0, 200, 300 s/mm^2^, 2b = 0, 300 s/mm^2^, and 3b1 = 0, 100, 200 s/mm^2^

ADC values were then calculated for different *b* value combinations (3b2 = 0, 200, 300 s/mm^2^; 2b = 0, 300 s/mm^2^; 3b1 = 0, 100, 200 s/mm^2^) and statistically compared to the 4b reference (4b = 0, 100, 200, 300 s/mm^2^). Absolute ADC values of healthy liver parenchyma are shown in Fig. 1 and Table 2. Combinations 3b1, 3b2, and 2b resulted in significantly higher ADC values than those performed with four *b* values (*p*_3b2_ < 0.001; *p*_2b_ = 0.001; *p*_3b1_ < 0.001). The median overestimation of ADC compared to 4b is calculated with 0.015 × 10^−3^ mm^2^/s for 2b, 0.163 × 10^−3^ mm^2^/s for 3b2, and 0.791 × 10^−3^ mm^2^/s for 3b1.

**Table 2:** ADC values of healthy liver parenchyma in rats (*n* = 18) calculated from different *b* value combinations

Method	4b^a^	3b2^b^	3b1^c^	2b^d^
ADC interquartile range (10–3 mm^2^/s)	0.39	0.44	0.58	0.52
ADC median (10–3 mm^2^/s)	2.69	2.79	3.48	2.81
ADC minimum (10–3 mm^2^/s)	2.23	2.42	2.83	2.28
ADC maximum (10–3 mm^2^/s)	3.16	3.40	5.75	3.37

^a^4b = 0, 100, 200, 300 s/mm^2^

^b^3b2 = 0, 200, 300 s/mm^2^

^c^3b1 = 0, 100, 200 s/mm^2^

^d^2b = 0, 300 s/mm^2^

**Fig. 2:**
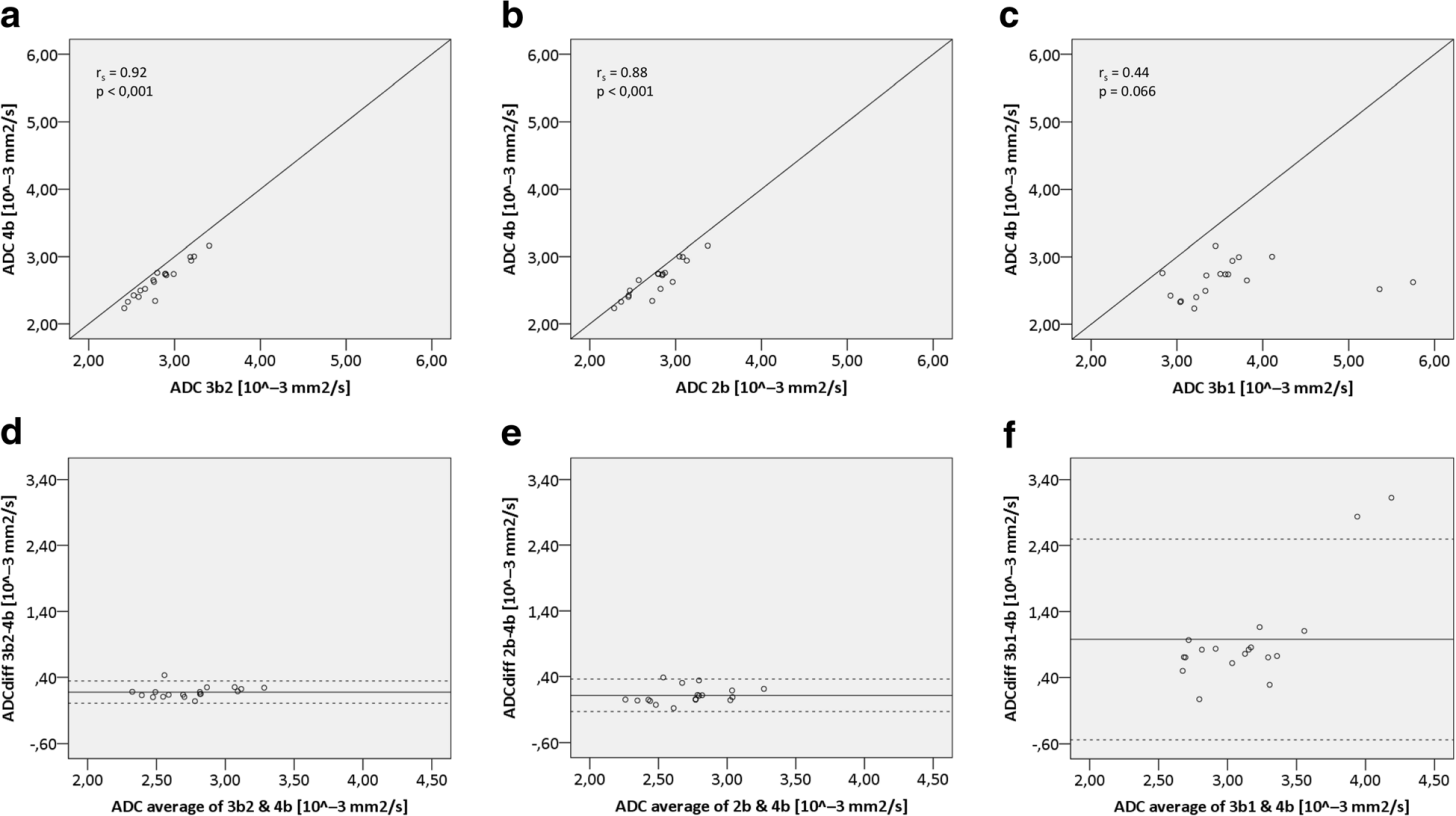
**a**–**c** Correlation plots of measurements using 4b (*b* = 0, 100, 200, 300 s/mm^2^) plotted against **a** 3b2 (*b* = 0, 200, 300 s/mm^2^), **b** 3b1 (*b* = 0,100, 200 s/mm^2^), and **c** 2b (*b* = 0, 300 s/mm^2^). *r*_s_ = spearman’s rank correlation coefficient. **d**–**f** Bland-Altman-Plots showing the differences between ADC measurements plotted against their corresponding average with 95% limits of agreement (dotted lines)

Correlation plots revealed high levels of correlation between 4b and 3b2 (*r*_s_ = 0.92; Fig. 2a) and 2b (*r*_s_ = 0.88; Fig. 2b), both correlations reaching statistical significance (*p* < 0.001). The correlation of 4b and 3b1 was clearly less distinct (*r*_s_ = 0.44; Fig. 2c) and did not reach the level of significance (*p* = 0.066).

Measurement with 3b2 resulted in a mean difference of 0.18 × 10^−3^ mm^2^/s with 95% limits of agreement 0.0074 × 10^−3^ and 0.34 × 10^−3^ mm^2^/s. Hence, the range between upper and lower limit of agreement amounted to 0.33 × 10^−3^ mm^2^/s (Fig. 2d). Data acquired using 2b showed the least mean difference of all three methods with 0.11 × 10^−3^ mm^2^/s and 95% limits of agreement − 0.13 × 10^−3^ and 0.36 × 10^−3^ mm^2^/s (Fig. 2e). The range here (0.49 × 10^−3^ mm^2^/s) slightly exceeded the previous one. Considerably different values were measured with 3b1, i.e., a mean difference of 3.1 × 10^−3^ mm^2^/s with 95% limits of agreement 2.3 × 10^−3^ and 4.0 × 10^−3^ mm^2^/s, resulting in a range of 1.7 × 10^−3^ mm^2^/s (Fig. 2f).

Based on the statistical analysis of *b* value combinations, we selected the 3b2 approach as the best agreement for performing DWI in the abdominal region (liver) with respect to correlation of ADC values, duration of scanning time, and scanner stability.

### Monitoring of HCC growth in rats

**Fig. 3:**
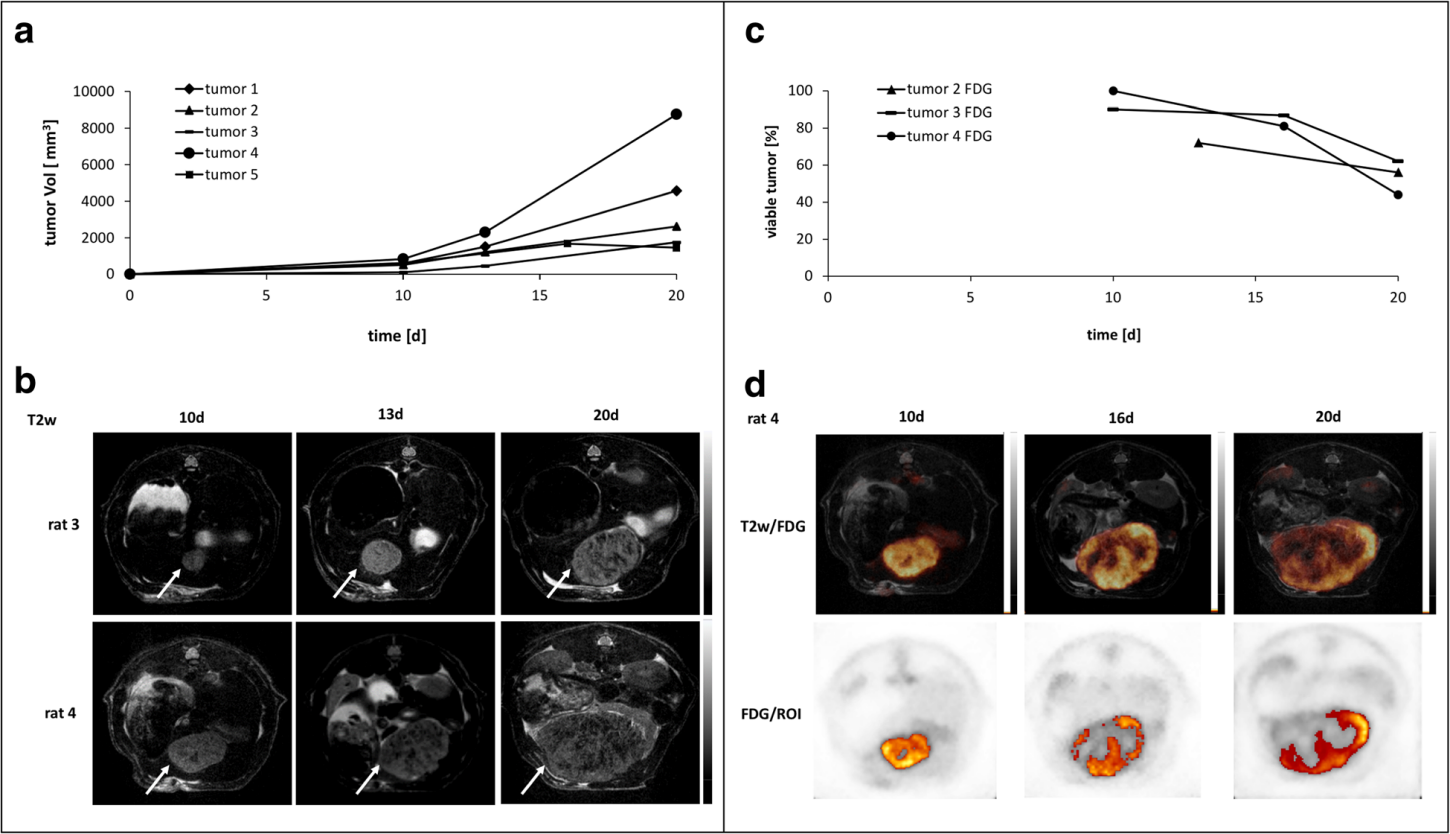
**a** Rate of HCC growth in rats. Tumor volume was measured after 10 and 20 days, as well as 13 days for tumors 1 to 4 and 16 days for tumor 5. **b** Representative T2-weighted images of rat 3 (tumor: white arrow) and rat 4 (tumor: white arrow) at three different time points revealing morphologic heterogeneity due to a different tumor growth rate. Viable tumor tissue is represented by hyperintense areas within the tumor, while degraded tumor tissue including necrotic areas is represented by hypointense areas. **c** The FDG-avid fractions of the tumor for three rats are shown. Decreasing values indicate increasing non-metabolically active tissue. **d** Fused FDG-PET/T2w tumor images of rat 4 (upper row) as well as threshold-based ROIs indicating viable tumor tissue for three different time points (d10, d16, d20)

Anatomical imaging based on T2-weighted images allowed a clear delineation of all tumors from the liver parenchyma and revealed an increasing heterogeneity of the tumor tissue during growth (Fig. 3b). Twenty days after tumor inoculation, tumor volumes ranged between 1717 and 6754 mm^3^. For tumor growth monitoring, animals were analyzed individually and served as their own control. One of the five tumors showed shrinkage by day 20 and was excluded from further image analysis. In T2-weighted sequences, viable tumor tissue appears as a hyperintense signal, while degraded tumor tissue results in rather hypointense signals. During the initial moderate growth phase until day 10 or 13, most tumors appeared solid with low levels of necrosis. Once tumor volumes expanded rapidly thereafter, the necrotic proportion increased, while viable tissue decreased (Fig. 3b).

**Fig. 4:**
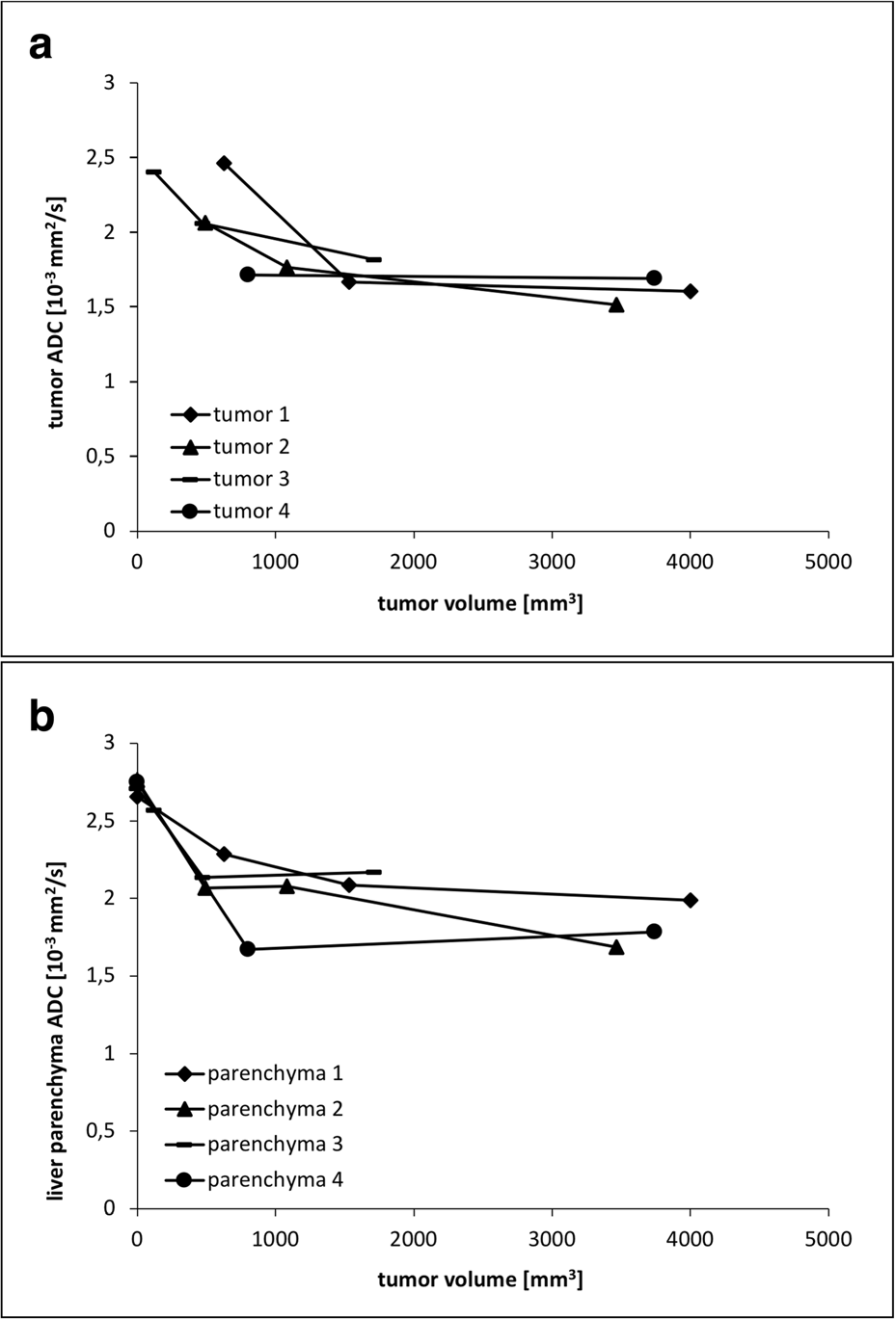
**a** ADC of the whole tumor as a function of the tumor volume. Measurements were performed after 10, 13, and 20 days for tumors 1 to 3 and, due to technical reasons, after 10 and 16 days for tumor 4. **b** ADC of the tumor surrounding liver parenchyma as a function of the tumor volume. Time points were the same as in Fig. 4a

ADC values of the individual tumors showed a drop during the initial growth in all homografts confirming an increase in cellular density. When tumors exceeded the size of 1500 mm^3^, ADC values remained on a plateau (around 1.6 × 10^−3^ mm^2^/s; Fig. 4a). One rat was measured only at two time points due to technical reasons. Interestingly, the ADC of the tumor surrounding liver parenchyma changed as well (Fig. 4b). During tumor tissue establishment (up to day 10), ADC of the liver parenchyma dropped clearly in all four homografted animals. When tumors increased in size, ADC of most parenchymas showed a plateau, while in one rat ADC values further decreased (Fig. 4b, parenchyma 2).

PET imaging revealed a decreasing percentage of the FDG-avid tumor fraction over time during tumor growth in all three animals (Fig. 3c and Table 3). All tumors showed an increasingly heterogeneous FDG uptake. In general, the center of the tumor did not accumulate FDG, indicating necrotic or degraded tumor tissue, while the tumor margin was still metabolically highly active (Fig. 3d). Fused FDG-PET/T2w images as well as threshold-based ROIs depicting viable tumor tissue are shown in Fig. 3d.

**Table 3:** Volumetry of FDG-PET in three tumor-bearing rats

Tumor	Day	Vol_tumor_ (mm^3^)	Vol_vital_ (mm^3^)	Viable tumor (%)
2	13	1532	1106	72
	20	3821	2151	56
3	10	0143	130	90
	16	1028	899	87
	20	1929	1200	62
4	10	0898	900	100
	16	3802	3090	81
	20	9272	4066	44

Vol_tumor_ = volume of the whole tumor measured by MRI; Vol_vital_ = FDG-avid tumor tissue defined as above a 45% threshold of the maximum tumor uptake

### Histopathology

**Fig. 5:**
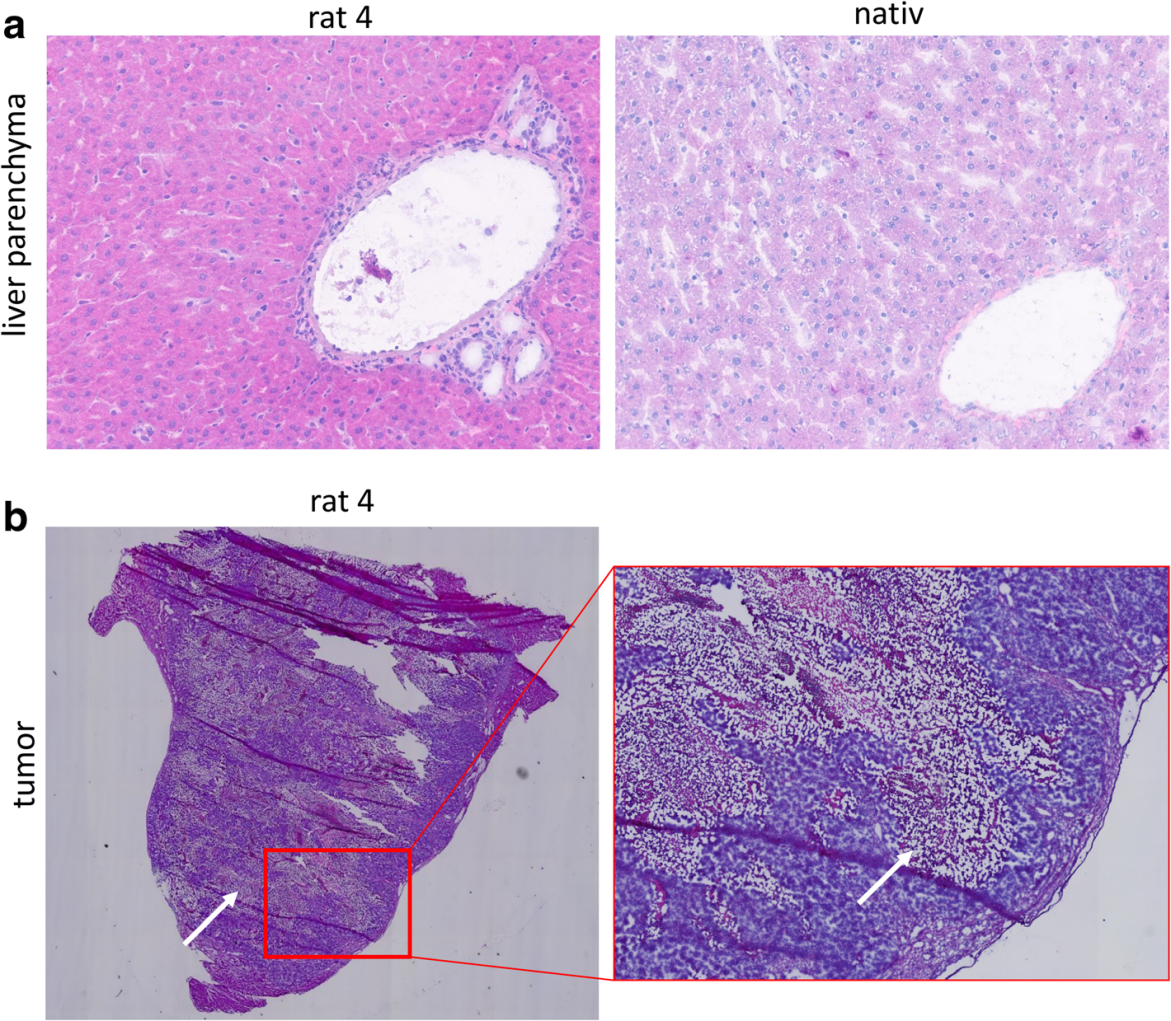
Hematoxylin and eosin (H&E) stained snap frozen tissue sections. **a** Native and tumor-bearing (rat 4) liver parenchyma. **b** Within tumor tissue large areas are necrotic (white arrow)

Histomorphology of liver sections was evaluated by H&E staining (Fig. 5a). Liver parenchyma of the native rat displayed normal morphology with no signs of inflammation or neoplasia, while liver parenchymas surrounding tumors showed varying levels of inflammation. While surrounding parenchyma from rat 1 had no inflammatory foci and showed only minimal inflammation in portal fields, the parenchyma surrounding the tumor in rat 3 displayed many foci of inflammatory cells that were partly necrotic, and all portal fields showed mild to moderate inflammation. Parenchymas that surrounded the tumors in rat 2 and 4 both showed comparable levels of inflammation of portal fields and numbers of inflammatory foci. Results are summarized in Table 4 along with numbers of inflammatory CD3^+^ T cells, CD163^+^ macrophages, and MPO^+^ neutrophils, and shown in Fig. 5 and Additional file 1: Figure S1. Tumor histopathology also confirmed large areas of tumor necrosis in accordance to tumor image characteristics (Fig. 5).

**Table 4:** Histopathology of liver parenchyma derived from healthy (native) and tumor-bearing rats, respectively

Specimen	H&E^a^		CD3^+^ (10 hpf)	CD163^+^ (10 hpf)	MPO^+^ (10 hpf)
	Inflammatory foci (0.25 cm^2^)	Hepatocytes (10 hpf)			
Native	0	2525	25	27	12
Tumor 1	0	2029	47	56	35
Tumor 2	3	3139	33	11	14
Tumor 3	16	3051	28	74	442
Tumor 4	2	2513	28	35	38

^a^H&E: hematoxylin and eosin

In order to evaluate whether hepatocytes were compressed by tumor growth or swollen due to injury or as a stress reaction of the microenvironment to tumors and/or inflammation, hepatocyte numbers were counted in 10 high power fields (× 200 magnification) (Table 4). While decreased numbers of hepatocytes indicate a cell swelling, increased numbers show compressed tissue.

## Discussion

Compared to strict anatomical MRI, DWI is considered to provide functional information. The resulting ADC values can be used to assess certain tissue characteristics, in particular tissue cellularity, that can function as a surrogate biomarker in the assessment of tumor therapy.

In this study, we established an optimized DWI protocol for the small animal nanoScan PET/MRI system equipped with a 1 T permanent magnet with respect to the known technical limitations. While initial DWI measurements using four *b* values including the maximum possible *b* value of 600 s/mm^2^ for this scanner resulted in gradient instability, most likely due to an overheated gradient system, we aimed for a robust sequence with less *b* values. The most promising magnitude of *b* values was identified in a phantom study and further tested to monitor biologic alterations during tumor growth of HCC lesions in a rat homograft tumor model.

A maximum *b* value of 300 s/mm^2^ finally used in this study is considered as a very low value. While brain DWI is usually performed with *b* values around 1000 s/mm^2^, for abdomen and liver lower maximum *b* values of 500–800 s/mm^2^ [16, 17, 22] are recommended, as higher values would increase the echo time (TE) as well as motion sensitivity and, thus, lower the signal-to-noise ratio [23] in organs that are prone to motion artifacts such as liver, e.g., due to respiration.

Technical limitations of the MRI hardware of the 1 T nanoScan PET/MRI scanner include that a maximum of four *b* values in the range of 0 to 600 s/mm^2^ is available only. According to the phantom studies, the set of four *b* values (4b = 0, 100, 200, 300 s/mm^2^) yielded the best results for this scanner with respect to ADC values for pure water published in literature. A disadvantage of this setup, however, was the long scanning time of 42 min, leading—in addition—to a decreased scanner stability when several animals were imaged in a row. We also noted that long scanning times resulted in overheating of the gradient system. Therefore, different setups for DWI were investigated focusing on shorter acquisition times. The highest level of correlation and lowest scattering was found for 3b2 (*b* = 0, 200, 300 s/mm^2^), resulting in a mean scan duration of 29 min compared to 42 min using the 4b set up. Using the 3b2 setup also allowed stable DWI imaging all day long, using even other sequences such as T1 and long T2w in between. However, in comparison to the 4b setup, a mean overestimation of ADC values of 7% was observed with the chosen parameters. Le Bihan et al. [24] described the influence of microcapillary blood flow when using low *b* values, e.g., from 0 to about 100 s/mm^2^ [12, 25–27]. Hence, using *b* values > 150 s/mm^2^ [28, 29] is necessary to avoid blood flow-corrupted ADC values.

In order to detect alterations in ADC values as an indicator for changes in biological processes during longitudinal studies, we considered the absolute ADC value (represented by the median (Fig. 1)) of less importance and focused rather on the precision of measurements (represented by the IQR (Fig. 1) and range between the 95% limits of agreement (Fig. 2)), because large scatter may mask minor changes in the values. Taken together, we recommend using the 3b2 setup (*b* = 0, 200, 300 s/mm^2^) for respiratory-gated DWI measurements in the abdominal region as the best setup with respect to accuracy, acquisition time, and performance stability of the scanner.

As a proof of concept, we applied the established DWI sequence for monitoring tumor growth in an orthotopic HCC rat homograft tumor model. Once tumors developed, an increasing cellular density of the tumor was represented by decreasing ADC values. The observed plateau of ADC values, once tumors exceeded a size of approximately 1500 mm^3^, resulted from balanced cell density and necrotic areas within the tumors as seen on T2-weighted images (Fig. 3b) and confirmed by histopathology (Fig. 5). These findings were paralleled by PET results revealing an increasingly heterogeneous FDG uptake in the growing tumors and a drop of the FDG-avid fraction of the tumor over time. Interestingly, the center of the tumors did not accumulate FDG, indicating necrotic or degraded tumor tissue, while the tumor margins were still metabolically active which can be best observed on fused FDG-PET/T2w images as well as by parametric threshold-based PET ROIs fused onto MR images. These FDG patterns are well-known in fast growing tumors where tumor growths is primarily limited to the tumor margins while the center becomes increasingly necrotic due to perfusion constraints. As demonstrated by fused images, metabolically active tumor areas on PET images can considerably differ from the actual total tumor volume identified best by T2w MRI. The sub-portion of non-metabolically active tumor mass may be a mix of necrosis, cells undergoing transition from metabolically active to inactive state, and areas of both. By performing both FDG-PET and anatomical and functional MRI, fused PET/MRI can improve tumor texture analysis and contribute to further characterization of tumor tissue providing different biological information.

In addition to the changes of the tumor ADC values measured over time, changes of the ADC values of the tumor surrounding liver parenchyma were also observed, indicating morphologic alterations of the liver parenchyma during tumor growth. A possible explanation for this observation is the interaction between tumor cells and surrounding tissue as an immunologic response. The observed decrease of the ADC values indicates a reduction in free water motion, which may be caused by infiltration of leucocytes, restricted sinusoids or swelling of hepatocytes due to inflammatory processes, all resulting in a reduction of the extracellular water-filled space. Taouli et al. [11] previously described lower ADC values in fibrotic and inflamed liver compared to healthy liver tissue. DWI has also been sensitive to inflammatory processes in other organs, e.g., in the lungs [9] and intestine [10].

Histopathologic findings in our study revealed varying levels of inflammation in the liver parenchyma of tumor bearing rats. As histomorphology is better preserved in formalin-fixed tissues, evaluation of H&E-stained cryo sections was impaired, and thus, it was difficult to differentiate between parenchymal changes caused by tumor growth and inflammation and artifacts resulting from fixation and histopathological processing. Therefore, we focused on an alternative but indirect method by counting the hepatocytes in 10 high power fields (Table 1). While lower numbers are indicative for swollen hepatocytes, higher numbers indicate a compression of the liver parenchyma. Our results revealed varying numbers of hepatocytes but no consistent alterations in all animals. As histopathology was only performed in single slices of each specimen, the immunohistochemical analysis in our study represents only a small snapshot of the total liver parenchyma surrounding the tumor. Therefore, further studies with improved histopathological approaches to confirm either inflammatory processes or other changes in the microenvironment and extracellular matrix are necessary to confirm and explain our observation of altered ADC values in the liver tissue adjacent to HCC tumor lesions.

## Conclusion

In conclusion, with respect to hardware restrictions of the 1 T nanoScan PET/MRI scanner, we established a feasible and reliable DWI imaging protocol for this system allowing to monitor biological changes in HCC liver tumors in a rat homograft tumor model over time. Focusing on the choice of *b* values for ADC calculation in the abdominal region, the use of three *b* values (0, 200, 300 s/mm^2^) delivered the best overall result when relevant parameters such as accuracy of the measurement, scanning time, respiratory gating, and performance stability of the system were taken into account. The optimized respiratory-gated DWI sequence presented here permitted high animal throughput and allowed to monitor morphologic changes during tumor growth, thus, proving the feasibility of DWI using this preclinical hybrid PET/MR scanner. However, each study needs to adapt the protocol according to their question, i.e., region of interest and system, to generate best possible results.

Furthermore, high-resolution anatomical MR imaging plus DWI in combination with FDG-PET provides additional and complementary information on tumor texture because DWI and FDG measure different patho-physiological aspects of tumor tissue. These different parameters of fused multiparametric PET/MR images may help to better characterize and understand tumor heterogeneity and changes of the adjacent host tissue during tumor growth in future research studies by providing different aspects of biological information on a molecular level within one imaging session.

## Additional file

Additional file 1: Immunohistochemic staining of liver parenchyma from tumor-bearing rats 1–4 and one healthy rat. (DOCX 2697 kb)

## Abbreviations

ADC: Apparent diffusion coefficient
ATCC: American Type Culture Collection
DWI: Diffusion-weighted imaging
EPI: Echo-planar imaging
FDG: 2-Deoxy-2-(^18^F) fluoro-d-glucose
FSE: Fast spin-echo
GRE: Gradient-echo
H&E: Hematoxylin and eosin
HCC: Hepatocellular carcinoma
HPF: High power field
IQR: Interquartile range
MRI: Magnetic resonance imaging
PET/MRI: Positron emission tomography/magnetic resonance imaging
ROI: Region of interest
SE: Spin-echo
T: Tesla
T1w/2w: T1-weighted/T2-weighted
TE: Echo time
