# Supplementary material for: Diffusion-weighted magnetic resonance imaging using a preclinical 1 T PET/MRI in healthy and tumor-bearing rats
Source: EJNMMI Res. 2019 Feb 22;9:21. doi: 10.1186/s13550-019-0489-6 (PMC6386759; doi:10.1186/s13550-019-0489-6)
Supplement: Supplementary file 1 — Immunohistochemic staining of liver parenchyma from tumor-bearing rats 1–4 and one healthy rat. (DOCX 2697 kb) [file 13550_2019_489_MOESM1_ESM.docx]

**Diffusion-weighted magnetic resonance imaging using a preclinical 1 T PET/MRI in healthy and tumor bearing rats**

Authors: Jakob Albrecht^1,2,3^, Dietrich Polenz^6,7^, Anja A. Kühl^5^, Julian M.M. Rogasch^1^, Annekatrin Leder^6,7^, Igor M. Sauer^6,7^, Magor Babos^8^, Gabor Mócsai^8^, Nicola Beindorff^4^, Ingo G. Steffen^1^, Winfried Brenner^1,2,4^, Eva J. Koziolek^1,2,3^

^1^ Department of Nuclear Medicine, Charité – Universitätsmedizin Berlin, Germany,

^2^ German Cancer Consortium (DKTK)

^3^ German Cancer Research Center (DKFZ) Heidelberg, Germany

^4^ Berlin Experimental Radionuclide Imaging Center (BERIC), Charité - Universitätsmedizin Berlin, Germany

^5^ Charité – Universitätsmedizin Berlin, Berlin Institute of Health, iPATH.Berlin – Immunopathology for Experimental Models, Core Unit

^6^ Department of Surgery, Campus Charité Mitte, Charité – Universitätsmedizin Berlin, Germany

^7^ Department of Surgery, Campus Virchow Klinikum, Charité - Universitätsmedizin Berlin, Germany

^8^ Mediso Ltd, Hungary

**Additional File**


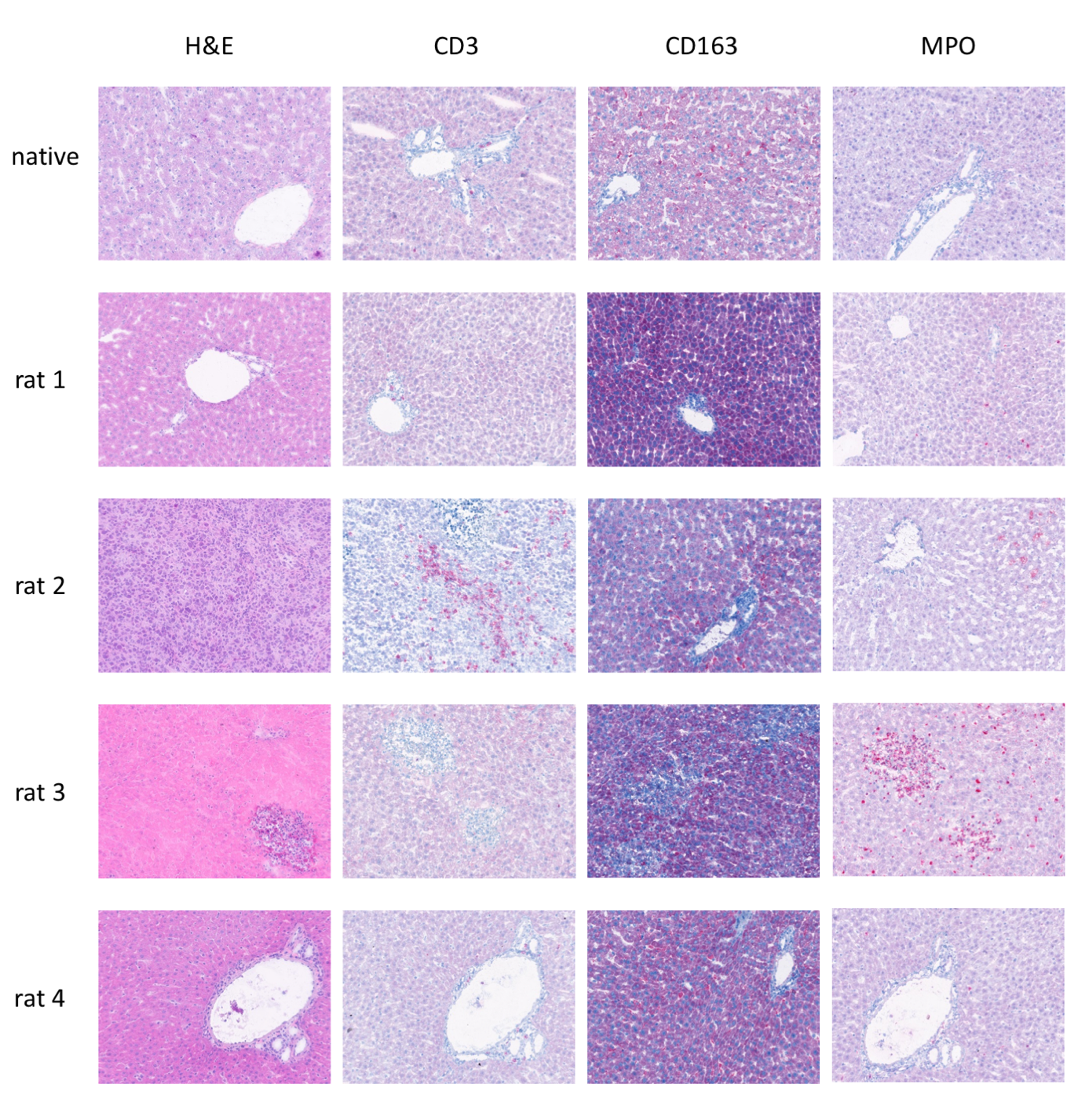


**Figure S1.** Immunohistochemic staining of liver parenchyma from rat 1 – 4 and one healthy rat, respectively.
